# Retraction: Land use land cover change as a casual factor for climate variability and trends in the Bilate River Basin, Ethiopia

**DOI:** 10.1371/journal.pone.0330113

**Published:** 2025-08-12

**Authors:** The PLOS One Editors

Following publication of [1], concerns were raised that this article contains a substantial degree of overlap with a previously published article [2] by the same authors, including almost all article text, reported methodology, equations, datasets and quantitative results.

On the basis of PLOS’ editorial assessment, the *PLOS One* Editors consider the extent of overlap is such that this article [1] constitutes a redundant publication. The corresponding author stated that prior to publication of [1] they requested to withdraw the accepted manuscript from *PLOS One* after the other article [2] was published; however, PLOS determined the request was not sent to a functional email address.

The *PLOS One* Editors retract this article [1] because, per our editorial assessment, it does not meet *PLOS One*’s second publication criterion [3].

SSB did not agree with the retraction. EV either did not respond directly or could not be reached.

The entire article [1] reports material similar to that published in [2] under a CC BY-NC-ND 4.0 license with some modifications. The earlier published article [2] was not cited in [1]. Source and licensing information for the map images in [1] was not provided in figure captions. Based on information provided during the manuscript submission process, Fig 1 is generated from OpenStreetMap and a shapefile obtained from the Ethiopian Geospatial Institute (EGI); Fig 3 is generated from freely available Landsat 5 MSS and Landsat 7 ETM+ provided by the U.S. Geological Survey archive; Fig 4 is generated from freely available Landsat 8 provided by the U.S. Geological Survey archive.
